# The Effects of a New Citrus Rootstock *Citrus junos* cv. Shuzhen No. 1 on Performances of Ten Hybrid Citrus Cultivars

**DOI:** 10.3390/plants13060794

**Published:** 2024-03-11

**Authors:** Wen He, Jiufeng Chai, Rui Xie, Yang Wu, Hao Wang, Yan Wang, Qing Chen, Zhiwei Wu, Mengyao Li, Yuanxiu Lin, Yunting Zhang, Ya Luo, Yong Zhang, Haoru Tang, Xiaorong Wang

**Affiliations:** 1 College of Horticulture, Sichuan Agricultural University, Chengdu 611130, China; hewen0724@gmail.com (W.H.); wangyanwxy@sicau.edu.cn (Y.W.); supnovel@gmail.com (Q.C.); 71444@sicau.edu.cn (Z.W.); limy@sicau.edu.cn (M.L.); linyx@sicau.edu.cn (Y.L.); asyunting@sicau.edu.cn (Y.Z.); luoya945@sicau.edu.cn (Y.L.); zhyong@sicau.edu.cn (Y.Z.); htang@sicau.edu.cn (H.T.); 2 Key Laboratory of Agricultural Bioinformatics, Ministry of Education, Chengdu 611130, China

**Keywords:** fragrant citrus, fruit quality, grafting, rootstock, tree vigor

## Abstract

The importance of rootstock in citrus production lies in its crucial role in determining tree growth, environmental stress tolerance, and fruit quality. *Citrus junos* Siebold ex Tanaka cv. Shuzhen No. 1, a recently developed rootstock, demonstrates excellent graft compatibility and abiotic stress tolerance. The objective of this study was to assess ten hybrid citrus cultivars grafted onto two *C. junos* rootstock selections, with the aim of determining the potential for industrial utilization of the new citrus rootstock. All graft junctions are mature and well established. Vigorous growth characterized all ten citrus cultivars on Shuzhen No. 1, with the largest tree’s height reaching 280.33 cm (Wogan scion) and the widest scion’s diameter being 67.52 cm (Chunjian scion). However, the scion-to-rootstock diameter ratio was the lowest at 0.62 (Chunxiang scion). *C. junos* rootstock selections significantly affected fruit weight (five of ten scions) and fruit color (seven of ten scions) but had negligible impact on peel thickness (nine of ten scions). Furthermore, rootstock type had a significant influence on fruit quality. In conclusion, our findings indicate strong graft compatibility between all scions and *C. junos* rootstocks, which can impact overall size and fruit quality. Based on these results, Shuzhen No. 1 is recommended as a valuable citrus rootstock.

## 1. Introduction

The genus *Citrus* encompasses a broad range of cultivated fruit crops of high global value [1]. Grafting, a commonly employed technique, involves merging a desired citrus scion with a compatible rootstock to propagate desired traits [2,3]. Rootstocks play a pivotal role in determining graft compatibility and scion vigor by influencing nutrient uptake, water absorption, disease resistance, and stress tolerance [4]. Selecting appropriate rootstocks is crucial, accounting for distinct environmental conditions [5,6]. Numerous studies have explored rootstock diversity, domestication, and their influence on plant vigor and stress responses [7,8,9]. 

Currently, rootstock selection for citrus production prioritizes compatibility and resistance; limited information, however, addresses rootstock impacts on performance of scion cultivars [10,11,12]. Despite the wide citrus grafting use, certain extensively employed rootstocks might still display graft incompatibility in the orchard, with manifestation taking years [13]. For instance, swingle citrumelo (*Citrus paradisi* × *Citrus trifoliata*), noted for biotic stress tolerance and enhancing scion fruit quality, exhibits incompatibility with specific sweet orange clones (*Citrus sinensis*) [14]. Trifoliate orange (*C. trifoliata*) serves as a commonly employed citrus rootstock due to its high resistance against various abiotic and biotic stresses [15,16]. However, it exhibits incompatibility with certain lemon (*Citrus limon*) and pummelo (*Citrus maxima*) cultivars [17,18]. With the dynamic shift in citrus cultivars, the need for rootstocks boasting compatibility and tolerance to multiple stresses hinders citrus industry growth.

Recently, hybrid citrus cultivars have been produced in southwest China, where the extensive calcareous purple soil type and the complex and variable climate pose huge challenges to citrus production [19]. The application of appropriate rootstocks in this region significantly impacts citrus scion resistance and fruit quality [20,21]. *Citrus junos* Siebold ex Tanaka cv. Ziyang Xiangcheng (CjZy), originating from southwest China, has gained widespread recognition as an iron-deficiency-, alkaline-, cold-, and acid-tolerant rootstock [22,23,24]. Nonetheless, for some specific hybrid citrus cultivars, CjZy cannot achieve entirely satisfactory indicators of fruit quality. For instance, in terms of the impact of rootstocks on the sugar content of Qingjian fruits, the effectiveness of CjZy was inferior to trifoliate orange [25]. Further, to obtain cultivars that perform better in the environment than the ones currently used, our previous citrus rootstock breeding program introduced a novel rootstock, *C. junos* Siebold ex Tanaka cv. Shuzhen No. 1 (CjSz), renowned for its tolerance to multiple stresses, such as flooding, alkaline, and freezing stress [26,27,28]. Meanwhile, it performs well as a rootstock for pummelo [29]. However, there is currently a lack of systematic research on the application of CjSz in practical production and its impact on tree growth and fruit quality. 

Currently, there is rapid progress in the updating and selection of scion cultivars. However, the development of appropriate rootstocks is lagging behind [30]. In this study, ten cultivars of hybrid citrus, appreciated by consumers and commonly planted in southwestern China, were selected as scions. This study investigates the performance of the hybrid citrus cultivars grafted onto two *C. junos* rootstock selections, offering insights for rootstock selection and hybrid citrus production.

## 2. Materials and Methods

### 2.1. Plant Materials

The trial was conducted in Sichuan Agricultural University’s orchard located in Chengdu, China (latitude 30°56′ N, longitude 103°65′ E, altitude 518 m), where water and fertilizer were used appropriately, and pest control was standardized. The climate is subtropical humid monsoon, featuring an annual mean temperature of 15.9 °C and 1012.4 mm precipitation. In January 2019, ten promising hybrid citrus cultivars with diverse genetic backgrounds were grafted onto one-year-old rootstock seedlings: *Citrus junos* Siebold ex Tanaka cv. Shuzhen No. 1 (CjSz) and *C. junos* Siebold ex Tanaka cv. Ziyang Xiangcheng (CjZy). The graft combinations in this study were prepared using the bud-grafting method. Table 1 illustrates the ripening time and origin of these hybrid citrus cultivars in the Sichuan region. The grafted seedlings were planted in purple soil with a pH range of 6.8 to 7.4, spaced at 2.0 × 2.7 m intervals.

### 2.2. Vegetative Growth Parameter of the Tree

Three healthy trees per graft combination, exhibiting consistent growth potential, were chosen for assessing tree growth parameters. Plant height, rootstock, and scion diameter, along with the scion-to-rootstock diameter ratio, were measured in December 2022. To investigate the sprouting in 2022, ten newly developed shoots were selected from each tree. Their length and diameter were measured during the cessation of growth for spring, summer, and autumn shoots. The scion and rootstock diameters were measured five centimeters above and below the grafting junction, respectively. The length of the new shoot was assessed from branch base to top bud. The new shoot diameter was determined at 3 cm above its base. 

### 2.3. Relative Chlorophyll Contents Determination

Relative chlorophyll content was measured by SPAD-502 Chlorophyll Meter Model (Konica Minolta, Kyoto, Japan). During the cessation of growth for spring, summer, and autumn shoots, 20 leaves from different new shoot types of each tree were selected to measure SPAD. The SPAD value of a leaf was determined by calculating the average SPAD values of its upper, middle, and lower parts.

### 2.4. Photosynthetic Rate Measurement

The leaf photosynthetic rates of different new shoot types were measured using a LI-6400 portable photosynthesis system (Li-Cor, Inc., Lincoln, NE, USA) on sunny mornings after growth cessation of spring, summer, and autumn shoots, respectively. Each tree contributed three biological replicates, with each replicate consisting of three healthy, intact, sun-exposed, disease-free functional leaves selected randomly.

### 2.5. Fruit Collection and Quality Parameters Assessment 

**Figure 1:**
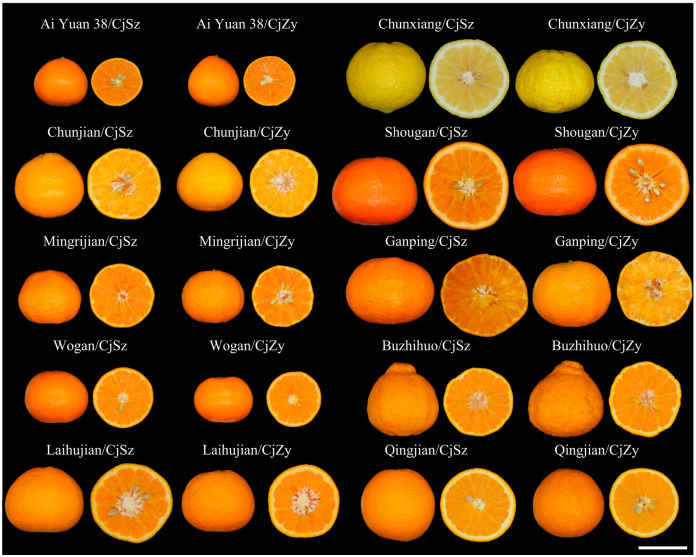
The appearance of fruits of ten hybrid citrus cultivars grafted onto two *C. junos* rootstocks. CjSz: *Citrus junos* Sieb. Tanaka cv. Shuzhen No. 1, CjZy: *C. junos* Sieb. Tanaka cv. Ziyang Xiangcheng. Scale bar = 5 cm.

Between December 2022 and March 2023, mature fruits were harvested from various crown directions to assess fruit quality based on GB/T 8210-2011 standards [31] (Figure 1). Each biological replicate consisted of ten fruits per tree, and three trees were selected for each period of the biological repeat. External quality was observed in five fruits from each tree, while the remaining fruits were stored at −20 °C for internal quality assessment. Fruit weight (FW) was measured. The horizontal and vertical diameters of fruits and equatorial peel thickness (PT) were measured directly using a Digital Vernier scale (Deli, Ningbo, China). The ratio of horizontal to vertical diameter defined the fruit shape index (FSI). The fruit’s color was quantified on opposing equatorial sides at room temperature using a Konica Minolta Hunter Lab colorimeter by the CIE (The Commission Internationale de l’Eclairage) system, yielding L*, a*, b*, C* and H0 values. The citrus color index (CCI = 1000 × a*/L* × b*) gauged surface color variance. L* value represents the brightness of the color. a* value implies its location between green and red, with positive and negative numbers indicating red and green, respectively. b* value is an indicator for measuring whether it leans towards yellow or blue, with positive and negative numbers indicating yellow and blue, respectively. C* value is a chromaticity indicator that refers to the ratio of colored components to white components, where 0 represents no color and 100 represents a very bright color. H0 value is the hue angle, and different angles represent different colors.

### 2.6. Fruit Inner Quality Parameters Assessment 

The total soluble solids (TSS) and titratable acidity (TA) of the juice were measured using a digital refractometer (ATAGO, Tokyo, Japan) expressed as percentages. The filtered fruit juice was used for the determination of the ascorbic acid content and total sugar. Ascorbic acid (Vc) content was determined via titration with 2, 6-dichlorophenolindophenol sodium salt 0.08 g·L^−1^; 2, 6-dichlorophenolindophenol sodium salt was used to titrate a mixture of 0.5 mL fruit juice and 4.5 mL 1% oxalic acid. Total sugar (TS) was ascertained using the sulphate–anthrone method. Diluted fruit juice and sulphate–anthrone solution were placed in the test tube and cooled naturally to room temperature, after keeping them in a thermostatic water bath at 95 °C for 10 min. Finally, the absorbance of the reaction solution was measured at the wavelength of 620 nm.

### 2.7. Data Processing and Analysis

Data were processed using Microsoft Excel 2020. The significance of differences among the data were assessed by using Software SPSS v.22.0 (SPSS-IBM, Corp., Armonk, NY, USA), with the following metrics: independent samples, *t*-test, two-tailed test, and a statistical significance level of *p* = 0.05. Significance was tested for indicators of the same scion with different rootstocks. The correlation matrixes of the different variables were visualized and conducted by the Pearson method using the OmicShare tools v.1.0 (https://www.omicshare.com/tools (accessed on 10 October 2023).

## 3. Results

### 3.1. Tree Growth

**Figure 2:**
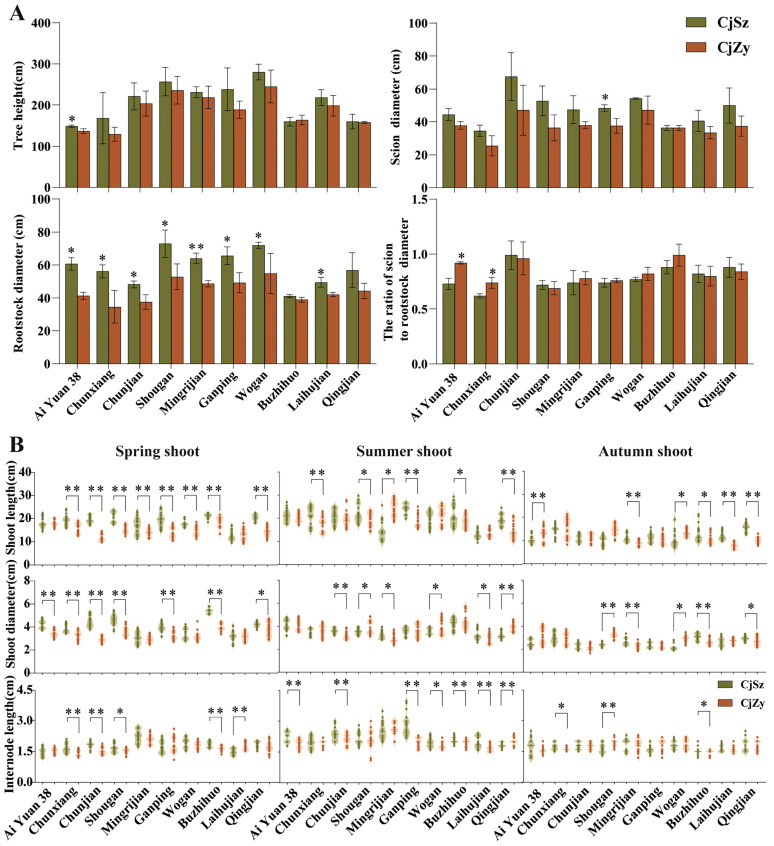
Effects of different rootstocks on tree growth of ten citrus scions. (**A**) Tree height, scion diameter, rootstock diameter, and the ratio of scion-to-rootstock diameter. Data were collected in December 2022. (**B**) Shoot length, diameter length, and internode length of spring, summer, and autumn shoots. Data were collected in 2022. The horizontal coordinates of the graph represent scions. The different colors represent scions grafted on different rootstocks. Significance was tested for indicators of the same scion with different rootstocks. A single asterisk (*) indicated significant differences at *p* < 0.05 and double asterisks (**) indicated significant differences at *p* < 0.01.

Tree height, shoot length, and diameter, along with stem thickness above and below the graft joint were measured in 2022, when all grafts were approximately four years old (Figure 2). Remarkable tree vigor was displayed by the scion Wogan and the rootstock CjSz in comparison to other scion–rootstock combinations (Figure 2A). This particular combination not only exhibited the largest tree height at 280.33 cm but also showed the second largest scion diameter at 54.23 cm. The highest scion-to-rootstock diameter ratio, indicating successful grafting fusion and harmonious growth, was observed on Chunjian grafted onto CjSz and Buzhihuo grafted onto CjZy, both showcasing a ratio of 0.99 (Figure 2A). Conversely, when Chunxiang was grafted onto CjSz, the scion-to-rootstock diameter ratio reached its lowest value. Despite this minimum value of 0.69, the findings suggested successful grafting compatibility (Figure 2A).

Compared to cultivars grafted on CjZy, most cultivars grafted on CjSz exhibited significantly higher growth potential in terms of stem length, stem thickness, and internode length (Figure 2B). Notably, trees of the cultivars AiYuan 38, Chunxiang, Chunjian, Shougan, Ganping, Buzhihuo, and Qingjian, when grafted on CjSz, had significantly larger spring shoot diameters than CjZy, with diameters of 4.23 cm, 3.83 cm, 4.49 cm, 4.61 cm, 3.94 cm, 5.39 cm, and 4.20 cm, respectively (Figure 2B). The use of CjSz rootstock also increased the spring shoot length of the cultivars Chunxiang, Chunjian, Shougan, Mingrijian, Ganping, Wogan, Buzhihuo, and Qingjian, compared to CjZy (Figure 2B). Regarding autumn shoot growth indicators, the diameters of Mingrijian, Buzhihuo, and Qingjian on the CjSz rootstock were significantly larger than that of CjZy. Additionally, the shoot lengths of Mingrijian, Laihujian, Buzhihuo, and Qingjian were significantly thicker than that of CjSz (Figure 2B).

### 3.2. Leaf Greenness and Photosynthetic Capacity

The highest SPAD value at 85.27, an indicator of leaf greenness, emerged in leaves obtained from the summer shoot, which were grafted on CjZy, specifically Buzhihuo and Chunxiang. Conversely, leaves grafted with Chunjian onto CjZy displayed the lowest SPAD value at 59.91 (Table 2). The use of CjSz rootstock positively influenced the leaf SPAD value of summer shoot of Chunjian and Ganping, with values of 73.25 and 80.31 (Table 2). Based on the leaf performance during the autumn shoot cessation, there was a significant difference in leaf greenness of scions grafted on CjSz rootstock compared to CjZy, including Chunjian, Mingrijian, Ganping, Wogan, and Qingjian (Table 2). Overall, when the CjSz was used as a rootstock, the relative chlorophyll content of the leaves of the cultivars Mingrijian, Wogan, Chunjian, Buzhihuo, Qingjian, AiYuan 38, and Ganping exhibited a significant decrease. The rootstock choice significantly affected the net photosynthetic rates (P_n_) of the leaves of the grafted cultivars (Table 2). In spring shoots, CjSz rootstock led to elevated P_n_ in six hybrid citrus cultivars—Mingrijian, Laihujian, Wogan, Ganping, Chunjian, and Buzhihuo—compared to CjZy. This underscored CjSz’s role in bolstering photosynthetic efficiency for these citrus cultivars in spring. Throughout summer shoots, all citrus cultivars displayed similar photosynthetic capacities, regardless of their rootstock. For autumn shoots, the P_n_ value was notably higher in Ganping grafted onto CjZy than when grafted onto CjSz, and higher in AiYuan 38 grafted onto CjSz than when grafted onto CjZy (Table 2). In autumn shoots, Ganping cultivar’s P_n_ value significantly exceeded when grafted onto CjSz, compared to when grafted onto CjZy. Similarly, AiYuan 38 cultivar exhibited higher P_n_ value when grafted onto CjSz than onto CjZy (Table 2).

### 3.3. Fruit Exterior Quality

Fruit weight characterized all ten citrus cultivars on CjSz, with the largest average individual fruit weight reaching 281.43 g (Chunxiang scion) and the smallest mean fruit weight at 102.72 g (AiYuan 38 scion). When Ganping, Buzhihuo, and AiYuan 38 were grafted onto the CjZy, there was a noteworthy increase in fruit weight compared to when they were grafted onto the CjSz (Table 3). Furthermore, the results showed that significant differences occurred primarily in terms of fruit vertical horizontal diameter, showing a significant impact on fruit size. It is noteworthy that the fruit horizontal and vertical diameters of Chunjian and Qingjian, grafted to CjSz, had significantly higher horizontal and vertical diameters when compared to CjZy, while AiYuan 38 showed the opposite trend (Table 3). Ganping grafted on both studied rootstocks only demonstrated differences in fruit vertical diameter (Table 3). With respect to peel thickness, there was no significant difference in the performance of the nine cultivars on the two studied rootstocks, except when Ganping grafted on CjSz, whose combination of the fruits increased peel thickness by 37.6%, compared to CjZy (Table 3). 

The color of the fruit peel of ten cultivars grafted on two *C. junos* rootstocks is shown in Table 3. The lightness (L* value) in the fruit peel ranged from 54.73 (Shougan grafted on CjZy) to 69.23 (Chunxiang grafted on CjSz). It is worth noting that the L* values of the ten types of scions using CjSz was significantly higher than that using CjZy as the rootstock, indicating that CjSz can significantly improve the brightness of the fruit. Likewise, compared with CjZy as rootstock, the b* values of eight hybrid citrus cultivars grafted on CjSz were remarkably higher except for Wogan and Qingjian. In terms of a* value and the citrus color index (CCI), apart from four graft combinations that do not show significant differences, the remaining graft combinations displayed noticeable variations in color. The range of C* values were from 35.87 to 76.91, and the values of the fruit of eight of the ten scions grafted on the CjSz was significantly higher than that of the fruit grafted on the CjZy. This indicated that compared to CjZy, CjSz produced the more vibrant and saturated color. About the H0 values of the peel color, no significant differences were observed in only three of the ten citrus cultivars.

### 3.4. Fruit Inner Quality

**Figure 3:**
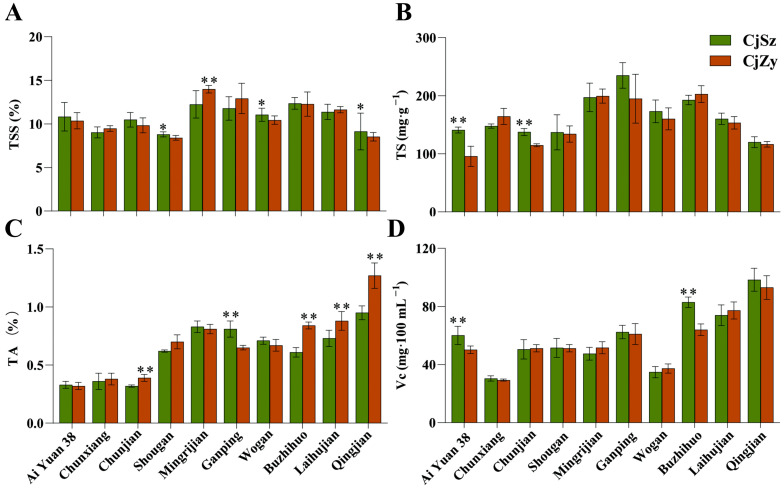
Effects of rootstocks on internal quality of ten citrus scions. (**A**) Juice total soluble solids (TSS); (**B**) total sugar (TS); (**C**) titratable acidity (TA); (**D**) ascorbic acid (Vc) content. The horizontal coordinates of the graph represent scions. The different colors represent scions grafted on different rootstocks. Significance was tested for indicators of the same scion with different rootstocks. A single asterisk (*) indicate significant differences at *p* < 0.05 and double asterisk (**) indicate significant differences at *p* < 0.01.

Variations in sugar and acid content are shown in Figure 3. Due to differences among cultivars, the range of titratable acids varied significantly, with a maximum value of 1.27% and a minimum value of 0.32%. The CjZy rootstock resulted in higher acid content for Laihujian, Chunjian, Buzhihuo, and Qingjian, compared to grafting onto the CjSz. Conversely, Ganping displayed elevated acid content when grafted on CjSz, as opposed to the CjZy. However, inconsistencies were observed in Vc content and TA content (Figure 3C,D). The Vc content of Buzhihuo and AiYuan 38 was higher when grafted on the CjSz rootstock, compared to CjZy (Figure 3D). Regarding TSS content comparison among different graft combinations, the positive impact was shown when CjSz rootstock was used, where there was an increase of 5.93%, 4.64%, and 6.91% in the TSS content of the fruits of the Wogan, Shougan, and Qingjian, respectively, compared to CjZy. In terms of TS content, except for AiYuan 38 and Chunjian, there was no significant difference between the other eight grafting combinations.

### 3.5. Correlation among the Parameters

**Figure 4:**
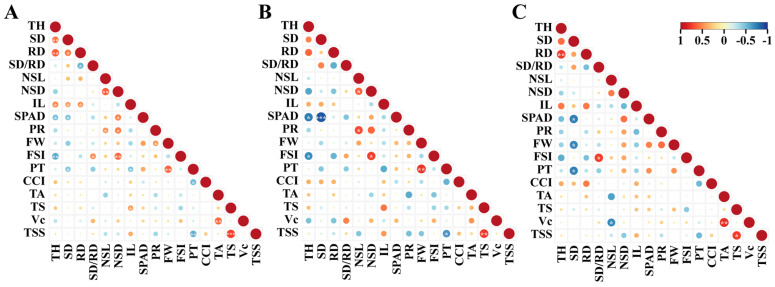
Correlation analysis of the parameters on horticultural characteristics. (**A**) Two rootstocks overall. (**B**) CjSz. (**C**) CjZy. TH, tree height; SD, scion diameter; RD, rootstock diameter; SD/RD, the ratio of scion-to-rootstock diameter; NSL, new shoot length; NSD, new shoot diameter; IL, internode length; SPAD, SPAD value; PR, photosynthetic rate; FW, fruit weight; FSI, fruit shape index; PT, peel thickness; CCI, citrus color index; TA, titratable acid; TS, total sugar; Vc, vitamin C; TSS, total soluble solids. A single asterisk (*) indicates statistically significant difference (*p* < 0.05), double asterisks (**) indicate highly statistically significant difference (*p* < 0.01) and three asterisks (***) indicate *p* value less than 0.001.

In the context of the overall values of ten citrus cultivars grafted onto two *C. junos* rootstock selections, an extremely significant correlation of 0.74 between total sugar (TS) and total soluble solids (TSS). This correlation was also observed for individual scion cultivars, with CjSz at 0.78 and CjZy at 0.76, respectively (Figure 4). Likewise, a significant correlation was noted between scion diameter (SD) and SPAD value in all graft combinations, for CjSz and CjZy, with respective coefficients of −0.47, −0.88, and −0.75 (Figure 4). 

Tree height (TH) showed extremely significant correlation with SPAD value and fruit shape index (FSI) at −0.75, −0.69, −0.48, and −0.58 in CjSz and all graft combinations, respectively (Figure 4). Similarly, new shoot length (NSL) exhibited an extremely significant correlation with new shoot diameter (NSD) and photosynthetic rate (PR) in CjSz and all graft combinations (Figure 4). The thickness of fruit peels (PT) demonstrated a negative correlation with TSS, with an extremely significant coefficient of −0.66 in CjSz, and significant coefficients of −0.57 in overall values, but insignificance in CjZy at −0.51 (Figure 4).

## 4. Discussion

The significant influence of rootstock on plant growth, photosynthesis, and fruit quality is widely acknowledged [32,33]. Therefore, it is crucial to ascertain the interactions between different commercial cultivars and rootstocks in specific regional environments, providing a new perspective for rootstock selection and citrus production. *Citrus junos* Siebold ex Tanaka cv. Shuzhen No. 1 (CjSz), a new citrus rootstock, has proven reliable against various abiotic and biotic stresses [26,27,34].

Graft joint assessments reveal no noticeable stem swelling, indicating strong graft compatibility between the scions and *C. junos* rootstocks (Figure 2A), consistent with previous findings [19]. Compared to the use of CjZy as the rootstock, most combinations grafted on CjSz had excellent performance in the length, coarseness, and internode length of spring, summer, and autumn shoots (Figure 2B), consistent with previous findings that CjSz had stronger tree vigor as the rootstock [35]. Previous studies have shown that the influence of rootstocks on scions also affects the leaves, causing significant differences in the content of chlorophyll and nutrients in scion leaves, thereby affecting leaf photosynthesis [36]. In addition, the higher photosynthetic capacity of fruit trees is the foundation for excellent quality. In this study, the same scion grafted onto the two rootstocks exhibited varied chlorophyll contents and photosynthetic capacities in leaves (Table 2). Noticeably, the CjSz rootstock led to higher net photosynthetic rates (P_n_) in spring shoots of six hybrid citrus cultivars compared to CjZy (Table 2), consistent with previous conclusion that CjSz rootstock caused an up-regulated photosynthetic capacity [29]. These differences may stem from varying root-borne resource supplies, such as water and minerals, to the shoots [37,38].

The rootstock has a significant impact on fruit external quality [39]. Notably, our results indicate higher fruit weights for Ganping, Buzhihuo, and AiYuan 38 grafted on CjZy compared to CjSz (Table 3). Similarly, compared to Hongju (*C. reticulata* Blanco) and trifoliate orange rootstocks, CjZy-based grafts yield a significantly higher average weight per fruit. Fruit size, an important quality characteristic of citrus fruits, determines their popularity in the fresh fruit market [40]. The influence of citrus rootstocks on the scion fruits size may stem from differences in various pathways that regulate fruit size. In the work of Liu et al. [41], it was proposed that the higher concentration of abscisic acid may inhibit the synthesis of growth-promoting hormones and hinder the growth and cell expansion of the Shatangju/trifoliate orange fruit. In our study, Chunjian and Qingjian grafted on CjSz exhibited significantly larger fruit horizontal and vertical diameters than those on the CjZy, while AiYuan 38 showed the opposite trend (Table 3). This provides a research basis for exploring the intrinsic mechanism of citrus rootstock influence on fruit size. Fruit color, a subjective attribute, to a certain extent determines consumers’ purchasing desire and is frequently associated with the ripeness or taste of fruits, affecting commercial activities in the market [42]. In this study, observable color differences were identified between two *C. junos* when grafted with the same scion (Table 3), providing valuable insights into genetic variations and underlying physiological mechanisms driving citrus fruit development [43]. Moreover, it is worth noting that the brightness (L*) of all types of scions using CjSz was significantly higher than CjZy. 

The quality and taste of citrus fruits, and subsequently consumer preferences, are heavily influenced by sugar and acid contents in juice [44]. Prior research demonstrated higher soluble solids content in citrus scions grafted onto trifoliate orange rootstock compared to those grafted on CjZy. Similar to previous results, we found that Shougan, Wogan, and Qingjian grafted on CjSz rootstocks had higher total soluble solid content than grafted on CjZy (Figure 3A). It is worth noting that rootstocks have more of an effect on TA than that of TSS, which was inconsistent with the results in pummelo ‘Guanxi Miyou’ [29]. This may be due to the difference between the hybrid citrus and pummelo scions used in the two studies. The titratable acidity is not only used as an indicator of citrus juice quality, but also as a reference standard for judging the proper harvest time in production implementation [45]. In this study, the fruit pulp titratable acid content of the Chunjian, Buzhihuo, Laihujian, and Qingjian grafted onto CjSz were notably lower than that onto CjZy (Figure 3C). However, further research will explore the quality comparison of the same scion grafted on CjSz and trifoliate orange.

To sum up, although rootstock can affect scion growth and fruit quality, there were some scions in this trial that did not differ significantly after being grafted on two rootstocks. This may be due to differences in scion–rootstock interaction caused by different genetic backgrounds of the scion. In this study, CjSz can enable scions to have stronger sprouting ability, stronger tree vigor, and more sufficient nutrition, which is beneficial for plants to better cope with various pressures and environmental changes, thus achieving high-quality production. Our results support that CjSz is a rootstock that can be used in citrus production. 

## 5. Conclusions

Citrus rootstocks are the underground parts of grafting combinations that help crops adapt to both biological and abiotic conditions. However, the lagging status of citrus rootstock breeding affects the industry’s development. *Citrus junos* Siebold ex Tanaka cv. Shuzhen No. 1 (CjSz), a new citrus rootstock introduced in our previous breeding program, is renowned for its tolerance to multiple stresses. Compared to the commonly used rootstock CjZy, our study highlights the notable benefits of using CjSz rootstocks, leading to increased tree sprouting ability, tree vigor, and photosynthetic activity. The fruit produced by scions grafted onto CjSz exhibited acceptable physiochemical quality. In summary, our study provides valuable insights into the potential value of CjSz as a substitute for currently used rootstocks for hybrid citrus cultivars. These findings have important value for both citrus growers and breeders, effectively facilitating optimized cultivation practices of citrus hybrid cultivars and offering perspective for further rootstock breeding.

## Figures and Tables

**Table 1:** Background information about scions used in this study.

Genotype	Parents	Maturity Stage in Sichuan Area
Ai Yuan 38	*Citrus reticulata* Blanco × *C. reticulata* Blanco	October to December
Chunxiang	*Citrus tamurana* hort. ex Tanaka × *C.* Spp. hort. ex	December to January
Chunjian	*C. reticulata* Blanco × *C. reticulata* Blanco	December to January
Shougan	*C. reticulata* Blanco × *Citrus sinensis* (L.) Osbeck	December to February
Mingrijian	*C. reticulata* Blanco × *C. reticulata* Blanco	Late December to middle February
Ganping	*C. reticulata* Blanco × *C. reticulata* Blanco	Early January to early March
Wogan	*C. reticulata* Blanco × *C. reticulata* Blanco	Late January to early March
Buzhihuo	*C. reticulata* Blanco × *C. reticulata* Blanco	February to April
Laihujian	*C. reticulata* Blanco × *C. reticulata* Blanco	Middle February to April
Qingjian	*Citrus unshiu* Macf × *C. sinensis* (L.) Osbeck	March to April

**Table 2:** Leaf greenness and net photosynthetic rate among different graft combinations.

Graft Combinations	SPAD Value			Photosynthetic Rate (μmol·m^−2^·s^−1^)		
	Spring Shoot	Summer Shoot	Autumn Shoot	Spring Shoot	Summer Shoot	Autumn Shoot
AiYuan 38/CjSz	69.72 ± 2.99 a	82.74 ± 2.29 b	80.84 ± 6.87 a	8.52 ± 0.19 a	17.62 ± 1.29 a	16.73 ± 1.66 a
AiYuan 38/CjZy	62.85 ± 2.45 b	84.77 ± 2.42 a	80.76 ± 2.27 a	7.81 ± 0.67 a	16.47 ± 1.96 a	13.06 ± 2.31 b
Chunxiang/CjSz	74.45 ± 2.39 a	84.90 ± 2.89 a	75.98 ± 1.30 b	8.36 ± 0.96 a	16.14 ± 1.33 a	15.61 ± 2.80 a
Chunxiang/CjZy	66.78 ± 2.97 b	85.19 ± 2.70 a	78.00 ± 1.71 a	8.02 ± 0.71 a	15.51 ± 2.08 a	11.94 ± 2.22 a
Chunjian/CjSz	66.57 ± 2.36 a	73.25 ± 4.11 a	61.06 ± 6.41 a	8.57 ± 0.13 a	17.76 ± 1.46 a	15.60 ± 2.98 a
Chunjian/CjZy	59.88 ± 2.21 b	59.91 ± 4.57 b	51.27 ± 4.75 b	7.00 ± 0.84 b	16.31 ± 2.32 a	16.43 ± 1.56 a
Shougan/CjSz	67.35 ± 1.34 a	75.54 ± 2.23 a	72.98 ± 3.84 a	10.05 ± 1.13 a	17.97 ± 2.02 a	14.63 ± 342 a
Shougan/CjZy	64.97 ± 3.20 b	77.24 ± 3.47 a	74.74 ± 1.67 a	7.19 ± 1.01 a	18.03 ± 3.42 a	13.46 ± 2.99 a
Mingrijian/CjSz	62.70 ± 1.07 a	78.71 ± 3.34 a	78.94 ± 3.28 a	7.70 ± 0.04 a	10.30 ± 1.31 a	9.30 ± 2.09 a
Mingrijian/CjZy	64.06 ± 2.45 a	77.91 ± 1.60 a	76.42 ± 3.16 b	6.28 ± 0.10 b	10.50 ± 0.59 a	9.57 ± 3.02 a
Ganping/CjSz	66.24 ± 1.47 a	80.31 ± 4.11 a	79.18 ± 3.79 a	10.45 ± 1.06 a	18.36 ± 2.89 a	11.47 ± 1.93 b
Ganping/CjZy	67.80 ± 1.82 a	75.91 ± 4.20 b	67.29 ± 5.41 b	9.10 ± 0.45 b	17.69 ± 3.02 a	15.24 ± 1.65 a
Wogan/CjSz	60.81 ± 3.30 a	74.72 ± 2.51 a	74.77 ± 2.75 b	9.16 ± 0.31 a	11.66 ± 2.34 a	9.50 ± 1.36 a
Wogan/CjZy	60.08 ± 2.45 a	73.37 ± 3.49 a	70.38 ± 3.05 a	7.45 ± 0.47 b	10.04 ± 1.87 a	11.87 ± 2.06 a
Buzhihuo/CjSz	75.18 ± 3.20 a	82.58 ± 1.89 a	75.64 ± 6.21 a	10.60 ± 0.49 a	16.03 ± 1.57 a	11.42 ± 3.54 a
Buzhihuo/CjZy	73.04 ± 2.10 b	85.27 ± 2.82 a	75.62 ± 2.91 a	8.38 ± 1.26 b	17.09 ± 1.16 a	14.80 ± 3.10 a
Laihujian/CjSz	69.24 ± 3.44 a	79.33 ± 0.77 a	74.04 ± 6.25 a	8.17 ± 0.35 a	13.09 ± 1.84 a	9.93 ± 2.10 a
Laihujian/CjZy	64.39 ± 3.49 b	79.60 ± 2.82 a	77.89 ± 3.17 a	7.24 ± 0.35 b	13.41 ± 0.54 a	12.39 ± 1.90 a
Qingjian/CjSz	72.41 ± 2.05 a	77.42 ± 3.86 a	79.44 ± 3.46 a	7.12 ± 1.06 a	17.31 ± 0.49 a	12.77 ± 1.58 a
Qingjian/CjZy	66.04 ± 3.11 b	76.72 ± 2.61 a	77.35 ± 2.47 b	7.85 ± 0.28 a	16.75 ± 0.96 a	13.19 ± 2.34 a

Note: The results were presented as mean ± SD. The performance of the same scion on two rootstocks was compared using the *t*-test method (comparison between two rootstock–scion combinations). Different lowercase letters indicate significant differences at *p* < 0.05.

**Table 3:** Effects of different rootstocks on appearance quality of ten citrus scions.

Graft Combination	Fruit Weight (g)	Horizontal Diameter (mm)	Vertical Diameter (mm)	Fruit Shape Index	Peel Thickness (mm)	CCI
AiYuan 38/CjSz	102.72 ± 7.74 b	58.25 ± 2.38 b	59.65 ± 0.53 b	1.02 ± 0.03 a	2.00 ± 0.32 a	10.40 ± 1.29 b
AiYuan 38/CjZy	177.44 ± 27.85 a	62.57 ± 2.53 a	63.07 ± 2.56 a	0.97 ± 0.03 b	2.56 ± 0.96 a	12.81 ± 1.25 a
Chunxiang/CjSz	281.43 ± 22.73 a	87.68 ± 3.57 a	72.14 ± 3.42 a	0.83 ± 0.04 a	5.64 ± 0.62 a	0.84 ± 0.34 a
Chunxiang/CjZy	267.92 ± 18.62 a	86.49 ± 3.35 a	71.88 ± 3.57 a	0.83 ± 0.05 a	5.55 ± 0.24 a	1.32 ± 0.83 b
Chunjian/CjSz	158.73 ± 28.28 a	72.61 ± 6.14 a	66.87 ± 2.48 a	0.93 ± 0.03 a	2.31 ± 0.70 a	7.61 ± 0.49 a
Chunjian/CjZy	120.27 ± 6.22 b	64.30 ± 1.17 b	58.49 ± 1.80 b	0.88 ± 0.02 b	2.15 ± 0.18 a	8.32 ± 1.51 a
Shougan/CjSz	266.44 ± 34.66 a	87.35 ± 4.23 a	70.90 ± 3.31 a	0.84 ± 0.04 a	4.60 ± 0.45 a	10.41 ± 1.04 b
Shougan/CjZy	254.40 ± 17.03 a	87.35 ± 2.36 a	69.25 ± 2.26 a	0.80 ± 0.03 b	4.58 ± 0.40 a	13.32 ± 1.18 a
Mingrijian/CjSz	137.73 ± 23.54 a	67.99 ± 4.10 a	57.18 ± 4.01 a	0.84 ± 0.01 a	2.23 ± 0.56 a	7.79 ± 0.57 b
Mingrijian/CjZy	124.02 ± 6.21 a	64.23 ± 1.60 a	54.85 ± 1.75 a	0.85 ± 0.01 a	2.30 ± 0.41 a	10.98 ± 1.55 a
Ganping/CjSz	247.59 ± 22.61 b	85.83 ± 2.73 a	64.13 ± 2.73 b	0.75 ± 0.03 a	2.45 ± 0.49 a	9.92 ± 0.71 b
Ganping/CjZy	290.97 ± 27.24 a	88.90 ± 3.73 a	66.98 ± 2.64 a	0.77 ± 0.05 a	1.78 ± 0.31 b	12.72 ± 1.27 a
Wogan/CjSz	105.30 ± 6.69 a	61.98 ± 1.94 a	50.03 ± 3.11 a	0.81 ± 0.01 a	2.50 ± 0.46 a	6.30 ± 1.00 a
Wogan/CjZy	103.85 ± 12.83 a	61.18 ± 2.93 a	49.39 ± 1.28 a	0.81 ± 0.02 a	2.63 ± 0.12 a	7.01 ± 1.31 a
Buzhihuo/CjSz	212.79 ± 15.82 b	76.30 ± 2.72 a	74.07 ± 5.03 a	1.01 ± 0.10 a	3.02 ± 0.23 a	5.88 ± 1.10 a
Buzhihuo/CjZy	259.12 ± 27.84 a	81.40 ± 4.24 a	78.02 ± 1.04 a	0.92 ± 0.02 a	3.40 ± 0.89 a	6.28 ± 0.90 a
Laihujian/CjSz	220.76 ± 26.79 a	82.87 ± 4.38 a	65.22 ± 2.84 a	0.82 ± 0.02 a	3.95 ± 0.48 a	7.13 ± 1.31 a
Laihujian/CjZy	218.16 ± 15.65 a	81.75 ± 2.18 a	64.54 ± 4.07 a	0.80 ± 0.03 a	3.67 ± 0.28 a	8.14 ± 0.71 a
Qingjian/CjSz	230.43 ± 27.97 a	80.92 ± 3.61 a	70.35 ± 3.73 a	0.90 ± 0.01 a	3.41 ± 0.23 a	3.83 ± 0.73 b
Qingjian/CjZy	179.37 ± 16.84 b	73.63 ± 2.54 b	66.57 ± 4.01 b	0.91 ± 0.04 a	3.20 ± 0.20 a	5.92 ± 0.65 a

Note: The results are presented as mean ± SD. The performance of the same scion on two rootstocks was compared using the *t*-test method (comparison between two rootstock–scion combinations). Different lowercase letters indicate significant differences at *p* < 0.05.

## Data Availability

Data are contained within the article.
